# Present value of future health data: ethics of data collection and use

**DOI:** 10.2471/BLT.19.237248

**Published:** 2020-12-15

**Authors:** Ann Dulhanty

**Affiliations:** a Department of Entrepreneurship and Strategy, Ted Rogers School of Management, Ryerson University, 350 Victoria Street, Toronto, Ontario, M5B 2K3, Canada.

Valuing a resource based on its future potential, when the resource may be developed into knowledge, services or products that deliver great social and economic value, is a familiar practice to those who invest in early stage projects. Applying this principle, individual medical data may be found to have significant value, as many benefits will be derived from collections of individual data through the application of machine learning on extensive data sets. Therefore, the current value of individual medical data is both highly uncertain and potentially significant.

Ethical approaches to the collection, use and sharing of personal health data could consider the current and future value of this data. Stakeholders in this process include the individual, providers of medical care, public health authorities, government policy-makers and commercial concerns that collect and use medical information. Combining the increasing value of individual data (of which medical data are a valuable subset) with current practices in the collection and use of individual medical data suggests that all stakeholders should consider the right of the individual to understand the potential value of their medical information and who should benefit from this value. 

Many individuals know their personal data are valuable. This knowledge is the central premise behind free internet functionality, such as search engines, social media and maps. Users provide value through indications of their interests, allowing ads to be targeted to a presumably receptive population. Advertisers pay for user data, albeit indirectly. In the United States of America, there is a general acceptance, or at least acquiescence, that data privacy is traded for services.^1^

By extracting additional details from online behaviour, posts and general information on multiple platforms, and adding these details to the capacity of modern technology such as artificial intelligence, the nuances of individual behaviour increasingly provide valuable information. If genetic makeup, lifestyle and medical history are added to this information, individual data can be rich with information.

Globally, awareness of the value of personal data is rapidly advancing. Over half of the world’s countries had personal data policies in 2018, and more such policies are in draft stage. In parallel, individual awareness is growing, exemplified by concerns about information security.^2^ The recent introduction of the General Data Protection Regulation in Europe endorses the nature of data as a personal possession.

For years, commercial concerns have offered products to make an individual’s data more valuable. For example, fitness apps and nutrition apps increase the usefulness of activity and food intake data, allowing the user to extract personalized health knowledge from their data. As time goes on, the value of the knowledge generated has the potential to increase, as the analytical and predictive power of apps advance. More recently, for-profit start-up companies in the United States have emerged with the goal of providing individual users a means of securely selling their personal medical data. Different firms focus on specific areas of health, such as genomics, health care or disease history. An individual may one day be able to sell their data many times over to different buyers, or to the same buyer in different packages.

Individuals, governments and businesses acknowledge the value of personal data, and the associated right to control personal (including medical) data, as inherent in the fundamental right to privacy. This right has value to the individual, and is being made more valuable to third parties by advancing technology.

Medical researchers are familiar with the concept of paying access fees for third party statistical databases, so the value existing in a database of personal data is established. An indication of the perceived value that may be derived from medical data is valuations placed on companies emerging in this area.^3^ The market for medical data could be worth billions of United States dollars (US$), with identified data at a premium. An identified data set has greater value because it can be linked to other data sets and therefore provide more comprehensive information. Validation of the data set is also possible with identified individual sources. A higher-quality product has higher value.

Medical data can be a coveted commodity, a valuable resource on which firms base a competitive advantage to develop new medicines, diagnostics and interventions. Data sets can be proprietary, unshared assets.^4^ Value increases with competition. Free market principles may apply, such as sale to the highest bidder or offers of premium pricing for exclusivity.

However, individual data are not a traditional commodity. Such data defy the concept of supply and demand in that for many applications, the more data there are, the better – the value of the data set increases nonlinearly. This concept applies whether the data are from more individuals, whether more data are from the same individual or whether more data are of the same kind over time. A recent study summarizes this concept well in presenting a case that the value of data needs to be reassessed now that the ability to network, compile and analyse the data have advanced.^5^

Another recent analysis of how to calculate a value for personal data highlights that many questions have to be answered before this calculation can be accomplished, and suggests several ways to value data.^6^ A simple, valid approach considers the per-user revenues of a company that obtains all its revenue from user-directed advertising. According to this analysis, the value of one person’s data appears to be around US$ 10 per year for a social media platform. Many commercial and not-for-profit entities are interested in collecting and selling data sets built from amassed personal data. Considering the uncertainty of the future value of the personal data that constitutes these data sets, it is timely for these entities to consider ethical approaches to data collection and sharing, including the value of this property to the individual.

Conversely, personal health data are recognized in the broader health-care community as a commodity so valuable that ownership should not be restricted but instead shared freely among all stakeholders who could possibly benefit from the data.^7^^,^^8^ Most granting agencies and international concerns that support the advancement of human health not only encourage, but require, the sharing of study data. The justifications for the quality of the science and sharing the benefits with diverse populations are compelling. Arguably, individual data have little value per se, but have great value when transformed to knowledge.^8^ However, just as all raw ingredients have value to the makers of the final product, each individual’s data has value to third parties that use the data.

In medical research fields, significant efforts are directed to data privacy protection,^4^^,^^8^^,^^9^ and to best practices in informed consent when data might be shared with unrelated researchers in the future.^4^^,^^8^ Researchers, policy-makers and society in general could also ask whether individuals should have the right to reap the value of their own data or manage the data in a way that allows them to exercise their freedom to derive the value from their data.

An interesting theme in the literature is the obligation of the individual to transfer their personal data to the cause of scientific advancement, for the betterment of society. Indeed, many study participants are motivated to make their personal data available to other researchers by improving the situation of their peers.^10^ However, fairness requires that if individuals donate their valuable possessions, they should do so willingly and with full knowledge of the value of such possessions.

Attaching a measurable value to personal information is now possible because numerous businesses derive their revenues from such general information. Medical data may be even more valuable, as the products derivable from such data could be substantial, considering the revenues that could be garnered for treatments of previously intractable conditions. Compared with personal information, where the product is advertisement targeted to an individual, products developed from personal medical information have the potential to benefit many people at once. 

Unlike data used for marketing purposes, the value of personal medical data does not necessarily increase with personal income. All involved stakeholders must consider vulnerable populations such as young people, those having disease or with stigmatized conditions, who may have data of most interest but also be less able to make objective decisions about the value of their data. Additionally, there may be populations not as familiar with data use to whom the concept of the value of their personal data is not clear.^11^

Thus, in light of the uncertain but likely growing value of personal medical data, consideration should be given to informing individuals of its potential value before they consent to contribute their data. Re-consent could occur at frequent intervals to increase fairness and transparency. While individuals are responsible for understanding their personal rights, those who collect and use medical data, such as medical practitioners and researchers, public health offices, governments and commercial entities could make vulnerable populations aware of potential uses of their data. Those that stand to benefit most from data use have the most responsibility to do so. The value of personal medical information, or simple uncertainty as to the future value, should be clear to the individual. 

Further dialogue among stakeholders should help ensure individuals are treated fairly for the value of their personal information, while allowing distributive justice to be gained from the sharing of medical data.
